# α-Glucosidase Inhibitory Activity of Tsuan-Kan Tea in Different Solid-State Aging Models: Phytochemical Biotransformation, Volatile Profile, and Molecular Docking

**DOI:** 10.3390/foods15142535

**Published:** 2026-07-17

**Authors:** Yen-Chun Yang, Yi-Chan Chiang, Po-Yuan Chiang

**Affiliations:** Department of Food Science and Biotechnology, National Chung Hsing University, 145 Xingda Road, South Dist., Taichung City 40227, Taiwan

**Keywords:** solid-state aging, Tsuan-Kan tea, antioxidant capacity, anti-glycemic capacity, Molecular docking

## Abstract

Tsuan-Kan tea (TKT) is a solid-state-aged mixture of citrus and tea leaves. This study examined how aging temperature, humidity, and time affect the properties of TKT. Solid-state aging (SSA) enriched active ingredients (total phenol content: 1.25–2.51-fold; total flavonoid content: 1.29–2.52-fold; DPPH radical scavenging activity: 7.16–18.35-fold; ferric-reducing antioxidant power: 5.06–11.96-fold) by avoiding traditional processing losses. Flavanone glycosides (narirutin: 2.81 mg/g; hesperidin: 37.33 mg/g), 5-hydroxymethylfurfural (0.20 mg/g), and tea polyphenols (catechin: 109.80 mg/g; theaflavin: 1.82 mg/g) increased substantially under high-temperature and humidity. Fourier-transform infrared spectroscopy suggested that hydrothermal environments promoted glycosidic bond cleavage (1078 cm^−1^) and enhanced π–π stacking between polyphenols and flavanone glycosides, improving solubility. During SSA, aroma profiles shifted from fruity and floral to woody and aged. Partial least squares regression revealed that nonenzymatic browning products, particularly 5-HMF (variable importance in projection [VIP] = 1.806), strongly correlated with α-glucosidase inhibition. Although naphthalene exhibited statistical collinearity (VIP = 2.300) and theoretical binding affinity in molecular docking, its potential toxicity as a thermal processing byproduct designates it as a quality control marker for process intensity. Overall, SSA enriches functional phytochemicals (e.g., polyphenols and 5-HMF) via non-enzymatic pathways, providing preliminary *in vitro* evidence for developing functional beverages.

## 1. Introduction

Tea (*Camellia sinensis* L. O. Kuntze) originated in Asia and has a long history. Different tea-making techniques, degrees of fermentation, and tea varieties contribute to the rich and diverse flavors of the beverage. Oolong tea, a semi-fermented (30–60%) tea [1], retains numerous natural precursors present in fresh tea leaves. Oolong tea develops unique floral and fruity aromas through repeated withering and rolling during post-harvest processing [2]. In recent decades, tea has become increasingly globalized and is now considered a global functional beverage [3], with benefits such as regulating lipid metabolism and ameliorating cardiovascular disease and diabetes [4,5]. Consumers’ increased health awareness also drives the development of other tea products.

Tsuan-Kan tea (TKT), a traditional beverage of the Taiwanese Hakka community, is prepared using “Hutou Gan” (*Citrus aurantium* L.). The fruit is scooped out, filled with tea leaves, and processed using the traditional “nine-steam and nine-sun drying” technique, yielding a tea with a distinctive flavor and dark-brown appearance [6]. However, traditional TKT production faces serious challenges. The fruit is scarce and seasonal, and the traditional production process relies heavily on manual labor, making it difficult to integrate with modern industrialized, large-scale production, leading to the gradual decline of the craft. Therefore, developing alternative raw materials and improving production processes are the primary goals for developing the TKS industry. In contrast with Hutou Gan, oranges (*Citrus sinensis* (L.) Osbeck) are widely cultivated and have a stable yield. Oranges are usually eaten fresh after peeling or used for juicing [7]. They are rich in vitamin C, dietary fiber, carotenoids, and various flavonoids [8]. Thus, oranges possess multiple benefits, including antioxidant, anti-inflammatory, neuroprotective, and gastric protective properties [9,10]. Oranges are popular with consumers because of their sweet and sour taste and rich aroma, and they are also the most common raw material for processed citrus products.

Solid-state aging (SSA) involves nonenzymatic browning reactions, which lead to component conversion and secondary metabolic reactions between the peel and tea leaves, promoting flavor and functional component production [11]. SSA is a novel approach that regulates temperature and humidity to accelerate these transformations. Plant cell structures are easily disrupted under high-temperature and high-humidity conditions, promoting the release of polyphenols and aromatic precursors, which are further converted into free phenolic acids, enhancing antioxidant capacity [12,13]. Meanwhile, high water activity (Aw 0.6–0.8) accelerates nonenzymatic browning [14,15], deepening color, firming texture, and intensifying sweet-sour and aged notes [13].

During aging under acidic conditions, the Maillard reaction generates 5-hydroxymethylfurfural (5-HMF), which possesses antioxidant activity and the potential to inhibit α-glucosidase. These effects help stabilize blood sugar levels by regulating postprandial carbohydrate breakdown [16,17,18]. The hydroxyl group in the molecular structure of 5-HMF is the active site for scavenging free radicals, whereas the entire conjugated system of the furan ring, including its double bond and the linked aldehyde group, works together to stabilize the generated molecule, enhancing the antioxidant efficacy of 5-HMF [15]. Numerous studies have shown that phytochemicals and their secondary metabolites, such as polyphenols and flavonoids, may inhibit α-glucosidase and exert antioxidant and anti-inflammatory effects. Thus, such compounds could be applied to the development of functional foods [19,20,21].

Therefore, this study aimed to investigate five aging modes to prepare TKT by controlling for temperature, humidity, and time: hot-air drying (HAO), continuous aging (CAO), steam and room-temperature drying cycle (SRO), steam and hot-air drying cycle (SHO), and steam and constant temperature/humidity cycle (SCO). The physical properties (appearance, color, color difference, weight loss, water activity, pH, total soluble solids, browning degree, and morphology) were evaluated. Furthermore, the chemical composition, including volatiles, was determined using high-performance liquid chromatography and gas chromatography–mass spectrometry. In addition, phytochemical biotransformations were assessed using Fourier-transform infrared spectroscopy. Finally, the most promising inhibitors were screened using partial least squares regression (PLS-R) as ligands for the α-glucosidase docking simulation to confirm the biotransformed metabolite markers. This study serves as a comprehensive evaluation and guideline for the optimal development of SSA technology for functional TKT production. This work differs from previously reported SSA studies in five key ways: (1) it employs a composite two-ingredient matrix (citrus peel + oolong tea leaves) in which inter-ingredient phytochemical interactions are a subject of investigation; (2) five SSA models are compared systematically; (3) GC–MS volatile profiling of 106 compounds across the full aging trajectory is provided; (4) PLS-R chemometrics are applied to identify functional bioactive markers from the volatile dataset; and (5) molecular docking is employed to rationalize candidate–enzyme interactions mechanistically. The current work does not claim clinical efficacy; all functional data are derived from validated *in vitro* screening assays, and absolute IC_50_ determination with cellular validation is designated as the primary direction for future work.

## 2. Materials and Methods

### 2.1. Materials and Chemicals

Fresh oranges (FOs, *Citrus sinensis* (L.) Osbeck) were purchased from a fruit store in Taichung, Taiwan, and had an average weight of 150 ± 10 g. FOs were stored at −20 °C before pretreatment. Oolong tea leaves were procured from a tea shop in Taichung, Taiwan. All reagents and standards were sourced from Sigma-Aldrich (Burlington, MA, USA). The commercial TKT samples (CG) were purchased from a privately owned tea factory (Taoyuan City, Taiwan).

### 2.2. Preparation of TKT

The samples underwent a pretreatment. A circular opening was made at the top of the FOs, and the pulp was scooped out and deseeded. The pulp was then crushed and mixed evenly with an equal weight of oolong tea leaves before being filled back into the hollowed fruit. The opening was secured with cotton thread and toothpicks. After steaming for 2 h and pre-drying with hot air for 22 h, the samples were divided into five groups. The HAO group continued drying in a hot-air dryer (Model 3926TB, Excalibur Dehydrator Co., Sacramento, CA, USA) for 20 days. The CAO group was processed in a constant temperature and humidity machine (Model BTH80/-20, Firstek Co., New Taipei, Taiwan) at 55 °C and 75% RH for 20 days. The remaining three groups underwent cyclic treatments: following the initial steaming and pre-drying steps, they were subjected to SRO, SHO, or SCO for 3 days. Each cycle lasted 4 days, with a total of 5 cycles conducted over 20 days. The SSA process was independently replicated in three batches (*n* = 3) to ensure reproducibility across batches. For every treatment at each storage period (Days 0, 4, 8, 12, 16, and 20) within each batch, three independent TKT samples (oranges) were randomly selected for analysis. Samples were freeze-dried, ground, passed through a 40-mesh sieve, and stored at −80 °C until analysis.

### 2.3. Quality Characteristics of TKT

#### 2.3.1. Appearance

Photographs were acquired and analyzed using a digital monocular lens (Canon Model 450D, Tokyo, Japan) coupled with a microscope camera (Sony Model E3ISPM 20000KPA, Tokyo, Japan).

#### 2.3.2. Weight Loss Rate

The weight of the TKT samples at each time point was recorded, and the weight loss rate was calculated using Equation (1).

(1) $$Weight\;loss\;rate\left(\%\right)=\frac{\left(W_{1}-W_{2}\right)}{W_{1}}\times 100$$

where W_1_ is the weight after the pretreatment, and W_2_ is the weight after the different aging treatments.

#### 2.3.3. Aspect Ratio

The height and width of the TKT samples at each time point were measured and recorded, and the aspect ratio was calculated using Equation (2).

(2) $$Aspect\;ratio=\frac{H}{W}$$

where H and W are the height and width of the samples, respectively, at different aging times.

#### 2.3.4. Color Analysis

A color meter was used (Model NE-4000, Nippon Denshoku Industries Co., Tokyo, Japan). The instrument was calibrated using a standard white board (X = 92.81, Y = 94.83, Z = 111.71) and a zero box (X = 0, Y = 0, Z = 0) before measurements. The measurements were conducted under a standard illuminant C and a 2° standard observer with a 30 mm aperture, and no blooming time was applied to the powdered samples. The measurements comprised the lightness (L^*^), red/green coordinate (a^*^), and yellow/blue coordinate (b^*^). The color difference (*ΔE*) was calculated using Equation (3), with the fresh TKT sample as the comparison [13].

(3) $$\Delta E=\sqrt{{\left({L^{*}}_{1}-{L^{*}}_{0}\right)}^{2}+{\left({a^{*}}_{1}-{a^{*}}_{0}\right)}^{2}+{\left({b^{*}}_{1}-{b^{*}}_{0}\right)}^{2}}$$

where L^*^_0_, a^*^_0_, and b^*^_0_, are the lightness, red/green coordinate, and yellow/blue coordinate of TKT after pretreatment, and L^*^_1_, a^*^_1_, and b^*^_1_ are those values at each sampling time, respectively.

#### 2.3.5. Water Activity

The TKT samples were divided into two parts (peel and mixtures) and measured in triplicate at ambient temperature, using a water activity analyzer (Aqualab 3TE, Meter Group Inc., Washington, DC, USA).

#### 2.3.6. Field Emission-Scanning Electron Microscope (FE-SEM)

Following Lee and Lai [22], the TKT samples were mounted onto the platform using conductive double-sided tape. The sample surface was sputter-coated for 30 s at a 10-mA current, using a metal ion coater (Model JEC-3000FC, JEOL Ltd.,Tokyo, Japan). Subsequently, a vacuum was created, and the sample was observed under an accelerating voltage of 1 kV. The electron beam was generated in the vacuum, focused to a small diameter, and used to scan across the sample surface through electromagnetic deflection coils in the electron column.

#### 2.3.7. Fourier-Transform Infrared Spectroscopy Analysis

Following Chiang and Chiang [16], all TKT samples were examined using a Fourier-transform infrared spectrometer (FTIR; Nicolet 6700, Thermo Fisher Scientific Co., Waltham, MA, USA) equipped with an MCT infrared detector (Thermo Fisher Scientific Co., Waltham, MA, USA). Spectral scans were performed over a wavenumber range of 650–4000 cm^−1^, with a resolution of 2 cm^−1^ and a scan time of 30 s per spectrum.

### 2.4. Physical Characteristics of TKT

TKT dry powder (1 g) was weighed, mixed with 25 mL of deionized water, and extracted using an ultrasonic oscillator (Model DC600H, Yuantuo Technology Ltd., Taichung, Taiwan) for 30 min. The mixture was then centrifuged at 3820× *g* for 10 min to separate the solid residue and supernatant. The supernatant was collected for subsequent analysis and referred to as TKT water extract (TKTWE).

#### 2.4.1. pH Value

After three-point calibration of the pH meter (SP-2300, Suntex Instruments Co., New Taipei, Taiwan), triplicate measurements were performed on the TKTWE.

#### 2.4.2. Total Soluble Solids (TSS)

Triplicate measurements of the TKTWE were conducted using a portable refractometer (MASTER-M, ATAGO Co., Tokyo, Japan), with results expressed in degrees Brix.

#### 2.4.3. Browning Degree (A_420_)

Nam et al.’s [23] method was used. A volume of 200 μL of the TKTWE was loaded into a 96-well plate, and the absorbance was measured at 420 nm using a microplate reader (SPECTROstar Nano, BMG Labtech Co., Ortenberg, Germany). Deionized water was used as the blank control.

### 2.5. Chemical Composition Analysis of TKT

TKT dry powder (1 g) was weighed and mixed with 25 mL of 70% ethanol (*v*/*v*). The mixture was extracted using ultrasound for 60 min, followed by centrifugation at 3820× *g* for 10 min to separate the solid and supernatant. The supernatant was then passed through a 0.22 μm PTFE filter (Waters Co., Milford, MA, USA) before analysis. This extract was referred to as the ethanol extract of TKT (TKTEE).

#### 2.5.1. Analysis of Organic Acids

Following Scherer et al.’s [24] procedure, the organic acid content was analyzed using a pump coupled to an HPLC–UV system (Chromaster 5420, Hitachi Co., Tokyo, Japan) and a Mightysil RP-18GP Aqua column (250 mm × 5.0 μm, 4.6 mm i.d.). A mixed standard solution (1–0.03125 mg/mL) of oxalic acid, tartaric acid, malic acid, and L-ascorbic acid was prepared for external standard quantification (mg/g dry weight, DW). The mobile phase consisted of 0.02 M NH_4_H_2_PO_4_ buffer (pH 2.2 ± 0.2), at a flow rate of 1 mL/min. Detection was performed at wavelengths of 210 nm and 243 nm.

#### 2.5.2. Analysis of Polyphenols

Following Hsu et al. [12], the polyphenol content was analyzed using the pump coupled to the Chromaster 5420 HPLC–UV system (Model Chromaster 5420, Hitachi Co., Tokyo, Japan) and Mightysil RP-18GP column (250 mm × 4.6 mm i.d., 5.0 μm; Kanto Co., Tokyo, Japan). A mixed standard solution (0.5–0.0015625 mg/mL) containing narirutin, hesperidin, nobiletin, catechins, and theaflavin was prepared for external standard quantification (mg/g DW). The mobile phase consisted of 0.1% formic acid (*v*/*v*) (A) and 100% acetonitrile (B), with the following gradient: 0–5 min, 95% A; 5–20 min, 63% A; 20–25 min, 50% A; 25–30 min, 20% A; 30–35 min, 0% A (100% B); 35–40 min, 63% A; 40–45 min, 95% A. The flow rate was 0.8 mL/min, and detection was performed at wavelengths of 280 nm and 330 nm.

#### 2.5.3. Analysis of Maillard Reaction Products

Following Lee et al.’s method [25], the content of Maillard reaction products (MRPs) was analyzed using the pump coupled to the Chromaster 5420 HPLC–UV system and Mightysil RP-18GP column. A mixed standard solution (0.5–0.0015625 mg/mL) containing 5-HMF and furfural was prepared for external standard quantification (mg/g DW). The mobile phase consisted of 12% acetonitrile (*v*/*v*), at a flow rate of 1 mL/min, and detection was performed at a wavelength of 284 nm.

### 2.6. Functionality and Antioxidant Activity of TKT

#### 2.6.1. Total Phenol Content (TPC)

Following the method by Rojas-Ocampo et al. [26], gallic acid was used as the standard (2–0.125 mg/mL), and quantification was performed using the external standard method (mg gallic acid equivalent/g DW) (Figure S1). A volume of 70 μL of the TKTEE was mixed with 70 μL of the Folin–Ciocalteu reagent and 35 μL of 10% sodium carbonate (*w*/*v*), then reacted at ambient temperature in the dark for 30 min. Absorbance was measured at a wavelength of 735 nm.

#### 2.6.2. Total Flavonoid Content (TFC)

Following the method by Saeed et al. [27], quercetin was used as the standard (2–0.125 mg/mL), and quantification was performed using the external standard method (mg quercetin equivalent/g DW) (Figure S1). A volume of 10 μL of the TKTEE was mixed with 60 μL of deionized water and 30 μL of 5% sodium nitrite (*w*/*v*), then reacted at ambient temperature in the dark for 6 min. Subsequently, 25 μL of 2.5% aluminum chloride (*w*/*v*), 25 μL of 2% sodium hydroxide (*w*/*v*), and 50 μL of deionized water were added. After thorough mixing, the mixture was reacted for 15 min, and absorbance was measured at 510 nm.

#### 2.6.3. 2,2-Diphenyl-1-picrylhydrazyl (DPPH) Radical-Scavenging Capacity

Following Mareček et al.’s method [28], Trolox was used as the standard (1–0.0625 mg/mL), and quantification was performed using the external standard method (mg Trolox equivalent (TE)/g DW) (Figure S1). First, a 100 mM Tris-HCl buffer was prepared and adjusted to pH 7.4. Then, 10 μL of the TKTEE, 40 μL of the Tris-HCl buffer, and 75 μL of 0.5 mM DPPH were mixed thoroughly and reacted at ambient temperature in the dark for 30 min. Absorbance was measured at 517 nm.

#### 2.6.4. Ferric-Reducing Antioxidant Power (FRAP)

Following the method by Rumpf et al. [29], Trolox was used as the standard (1–0.0625 mg/mL), and quantification was performed using the external standard method (mg TE/g DW) (Figure S1). First, a 300 mM acetate buffer was prepared and adjusted to pH 3.6, along with 10 mM 2,4,6-tripyridyl-s-triazine (TPTZ) and 20 mM iron chloride. These three solutions were mixed at a ratio of 10:1:1 (*v*/*v*/*v*) to form the FRAP reagent. Then, 20 μL of the TKTEE was mixed with 150 μL of the FRAP reagent, and the mixture was incubated at 37 °C in the dark for 10 min. Absorbance was measured at a wavelength of 593 nm.

### 2.7. In Vitro α-Glucosidase Inhibition Assay

Following Yan et al.’s method [30], phosphate-buffered saline was prepared at pH 7.4. Acarbose (0.07 mg/mL) was used as the positive control group (100% inhibition), along with a blank group and sample groups for analysis. Each reaction mixture (50 μL of TKTEE and 100 μL of α-glucosidase) was mixed thoroughly and incubated at ambient temperature in the dark for 10 min. Afterward, 50 μL of *p*-nitrophenyl-α-D-glucopyranoside (*p*-NPG) was added and reacted for another 5 min. Finally, absorbance was measured at 400 nm, and the inhibition rate was calculated using Equation (4).

(4) $$\alpha -glucosidase\;Inhibitory\left(\%\right)=\left(1-\frac{A_{sample}-A_{sample\_blank}}{A_{control}-A_{blank}}\right)\times 100$$

where $A_{\mathrm{sample}}$ is the absorbance of the reaction mixture containing TKTEE, enzyme, and substrate; $A_{sample\_blank}$ is the background absorbance of TKTEE and substrate without the enzyme; $A_{\mathrm{control}}$ is the absorbance of the enzyme and substrate without TKTEE (representing 100% uninhibited enzyme activity); and $A_{\mathrm{blank}}$ is the absorbance of the buffer and substrate without the enzyme. Acarbose was measured under the same conditions as TKTEE to serve as a positive control reference.

### 2.8. Gas Chromatography–Mass Spectrometry Analysis

Dried TKT samples (3 g) were mixed with 5 μL of internal standard (cyclohexanol) and subjected to solid-phase microextraction (SPME), which was conducted in a 50 °C water bath, using a DVB/CAR/PDMS fiber assembly (Supelco, Inc., Bellefonte, PA, USA) for 1 h. After adsorption, the fiber was desorbed in a GC injection port at 240 °C for 15 min [31]. The analysis was performed using a GC HP 6890 (Agilent Technologies, Inc., CA, USA) coupled to an HP 5973 MSD detector equipped with a DB-WAX capillary column (60 m × 0.25 mm × 0.25 μm, Agilent, Palo Alto, CA, USA). The inlet temperature was 240 °C. Helium was used as the carrier gas at 1 mL/min in splitless mode. The temperature program began at 40 °C for 1 min, was increased by 3 °C/min to 150 °C, then increased by 5 °C/min to 240 °C, and then held at this temperature for 10 min. Retention indices were calculated using C5–C25 *n*-alkanes, and compound identification was based on the Wiley library. Quantification was based on the compound peak area and internal-standard peak area ratio.

### 2.9. Molecular Docking

Molecular docking simulations were performed using AutoDock Vina (ver. 1.2.2, Scripps Research, La Jolla, CA, USA) to evaluate the inhibitory potential of ligands against α-glucosidase [32]. The crystal structure of the receptor (UniProt ID: P53341) and the ligand structures were retrieved from UniProt and PubChem, respectively. Preprocessing was conducted in ChimeraX (ver. 1.10.1) by removing water molecules and adding polar hydrogens. A steric grid box of 80 Å × 80 Å × 80 Å with 0.05 nm spacing was centered on the active site to define the search space, determining the binding affinity and inhibitory effectiveness of the compounds.

### 2.10. Statistical Analysis

The solid-state aging process was independently replicated in three batches (*n* = 3). For each batch, three randomly selected TKT samples were treated as analytical replicates, and their mean values were used as a single data point for statistical analysis. All results were expressed as the mean ± standard deviation (*n* = 3). Statistical analyses were performed using one-way analysis of variance (ANOVA) in SPSS (version 12.0; IBM Co., Armonk, NY, USA). Significance was determined by conducting Duncan’s multiple range tests (DMRTs). Differences were considered statistically significant at *p* < 0.05. XLSTAT 2023.3.0 (Addinsoft Co., Long Island City, NY, USA) was used to perform agglomerative hierarchical clustering (AHC), principal component analysis (PCA), heat maps, and PLS-R.

## 3. Results & Discussion

### 3.1. Physicochemical Properties

**Figure 1:**
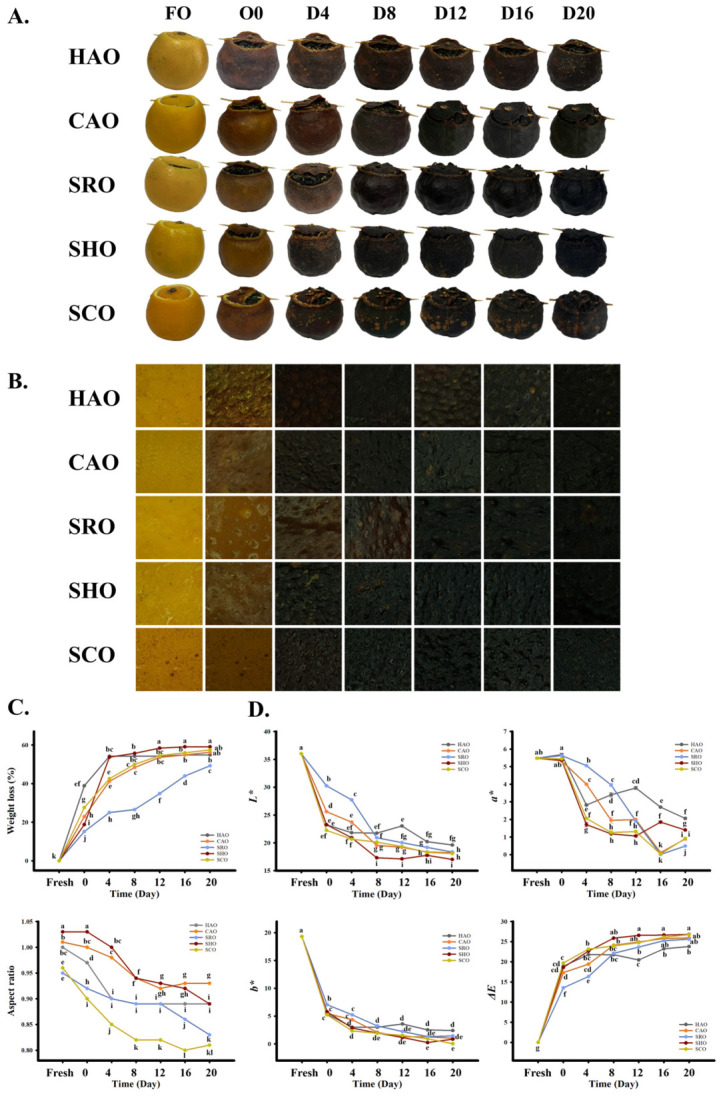
Appearance of Tsuan-Kan tea under different SSA treatments. (**A**) Appearance; (**B**) microstructure at 20× magnification; (**C**) weight loss rate and aspect ratio; (**D**) color analysis. Different letters (a–l) indicate significant differences in the same group (*p* < 0.05). HAO, CAO, SRO, SHO, and SCO represent the hot-air drying, continuous aging, steam and room-temperature drying cycle, steam and hot-air drying cycle, and steam and constant temperature/humidity cycle SSA treatments, respectively.

Appearance is a key factor in forming consumers’ first impressions of a product. Attributes such as color, gloss, and integrity influence subjective judgments of quality and affect purchasing intentions. Figure 1 illustrates the appearance of TKT after 20 days of being subjected to different SSA treatments. In Figure 1A, HAO exhibited rapid moisture loss from the peel during the early drying stage, leading to pronounced shrinkage and more prominent oil gland structures, compared with the other groups (Figure 1B) [33]. The drying rate decreased in the later stage because of the case-hardening effect (Figure 1C) [34,35]. Meanwhile, steaming disrupted cell walls in the three cyclic treatment groups (SRO, SHO, and SCO), resulting in the breakdown of polymers in the peel and tea leaves into smaller molecules, which provided more substrates for the browning reaction [36]. Among these groups, SRO showed a slower progression, as the other two groups were exposed to high temperatures for a prolonged time. Previous studies have indicated that thermal treatment facilitates nonenzymatic browning [37]. The aspect ratio (Figure 1C) decreased, indicating that the initially spherical shape (ratio close to 1) gradually flattened into a disc-like form, and the aspect ratio changed from 0.95–1.03 to 0.81–0.83, resembling the appearance of the commercial sample. In addition, lightness decreased from 36.02 to 17.02–19.62, whereas a and b values approached 0, indicating a transition toward dark-brown to black coloration. During the early aging period, *ΔE* increased rapidly from 0.00 to 13.55–19.70. In the later aging period, *ΔE* for HAO, CAO, SRO, SHO, and SCO increased to 23.79, 25.92, 25.61, 26.80, and 26.74, respectively (Figure 1D), consistent with the findings of Hsu et al. [12] and Chuang et al. [13].

The pH value decreased with increasing SSA duration, from 5.39 in the fresh state to 4.86–4.14 (*p* < 0.05). This drop is due to the formation of short-chain carboxylic acids during the Maillard reaction [38,39]. Notably, the three cyclic treatment groups (SRO, SHO, and SCO) exhibited lower pH values (4.38, 4.26, and 4.14, respectively). This observation can be attributed to cell wall disruption that promoted the release of polyphenols, followed by their oxidation or degradation into free phenolic acids, lowering the pH value. The TSS content decreased from 2.85 to 2.60–2.05 because of the consumption of reducing sugars during the Maillard reaction [40]. Finally, the water activity (Aw) in all groups dropped to below 0.65, inhibiting the growth of most putrefactive bacteria [41] (Table 1).

Nonenzymatic reactions (e.g., the Maillard reaction and thermal degradation) are primarily responsible for the substantial phytochemical transformations observed during the SSA process. As shown in Table 1, the Aw of all groups rapidly dropped to below 0.65 during the early aging stages. The low-moisture, high-temperature environment resulting from the combination of this Aw with the continuous or cyclical thermal treatments at 55 °C exceeds the growth threshold for most bacteria and fungi. Therefore, the cleavage of glycosidic bonds and the generation of secondary metabolites in this study are fundamentally attributed to hydrothermally induced nonenzymatic browning.

### 3.2. Organic Acid and Polyphenol Analysis

Organic acids are key contributors to the sour taste of fruits; they influence flavor and quality and are closely associated with health benefits. From a sensory perspective, organic acids play a decisive role in flavor perception and product acceptability [42]. Tartaric acid, malic acid, and ascorbic acid are the most abundant and important flavor compounds in citrus fruits. Thus, the concentrations of these three acids reflect changes in flavor quality during processing and serve as indirect indicators of product market potential. As shown in Table 2, the tartaric acid content decreased from 7.56 to 2.69–3.57 mg/g DW in all samples, except for SCO, where it slightly increased to 4.69–5.53 mg/g DW. This result suggests that tartaric acid leaches out during steaming and is lost during drying; however, the high-humidity environment in SCO allowed its retention [43]. The malic acid content increased in the hot-air drying groups (HAO and SHO) because of substrate concentration effects, but decreased in other groups because of degradation, dropping from 1.96 to 0.96–1.81 mg/g DW [44,45]. The ascorbic acid content increased after heat treatment, likely because abundant polyphenols, particularly catechins, acted as radical scavengers and reducing agents, regenerating oxidized dehydroascorbic acid back to ascorbic acid, increasing its overall content [46]. Chiang and Chiang [16] reported that ascorbic acid degradation yielded carcinogenic furfural; however, Maillard reaction product analysis in the current study detected no furfural, ensuring food safety.

Flavanone glycosides are the most abundant in citrus, with narirutin and hesperidin being marker compounds. By contrast, catechins and their oxidative polymers predominate in tea. Table 2 shows that the narirutin content increased across most groups, from 1.90 to 1.61–2.81 mg/g DW, all significantly higher amounts than in the commercial sample. Hesperidin first decreased and then increased in all groups except for SHO. Glycosylated flavanones are prone to hydrolysis during aging, yielding aglycones [47]. However, HPLC–UV analysis revealed a rebound in the flavanone glycoside content during the late stage of SSA. This phenomenon may be associated with hydrophobic interactions and aromatic π–π stacking between tea polyphenols and flavanone glycosides, leading to the formation of stable complexes. Such π–π stacking and hydrophobic interactions could enhance the apparent solubility and detectable levels of these complexes [48,49,50].

The total catechins were estimated as the sum of epigallocatechin gallate (EGCG), epigallocatechin (EGC), epicatechin gallate (ECG), epicatechin (EC), and catechin (C). EGCG predominates (25–40%) in oolong tea [1], followed by EGC (15–25%), ECG (10–20%), and EC (10–20%). By contrast, (+)-catechin typically indicates < 5%. Total catechins decreased from 117.87 to 78.98–94.60 mg/g DW, with SHO showing the greatest decline, although the catechin content was still significantly higher than that in the commercial sample (*p* < 0.05). The drop in the catechin content is likely due to their thermolabile nature, with EGCG being especially prone to ester hydrolysis into non-ester catechins (EGC, EC) or degradation into gallic acid and other phenolic acids [51]. The theaflavin content slightly declined from 1.78 to 1.62–1.70 mg/g DW. However, no theaflavins were detected in the commercial sample. Notably, in CAO, both catechin and theaflavin contents increased, from 102.03 to 109.80 mg/g DW and from 1.68 to 1.82 mg/g DW, respectively. This enhancement can be explained by the oxidative condensation of epicatechin and epigallocatechin gallate into theaflavins during SSA [52,53]. Moreover, the high-humidity environment provided by CAO facilitated the encapsulation of flavanone glycosides into the catechin dimer, improving solubility and contributing to the elevated concentrations of these compounds. Altogether, both total catechins and theaflavins were enhanced in CAO [48,51] (Table 2).

### 3.3. Browning Characteristic

**Figure 2:**
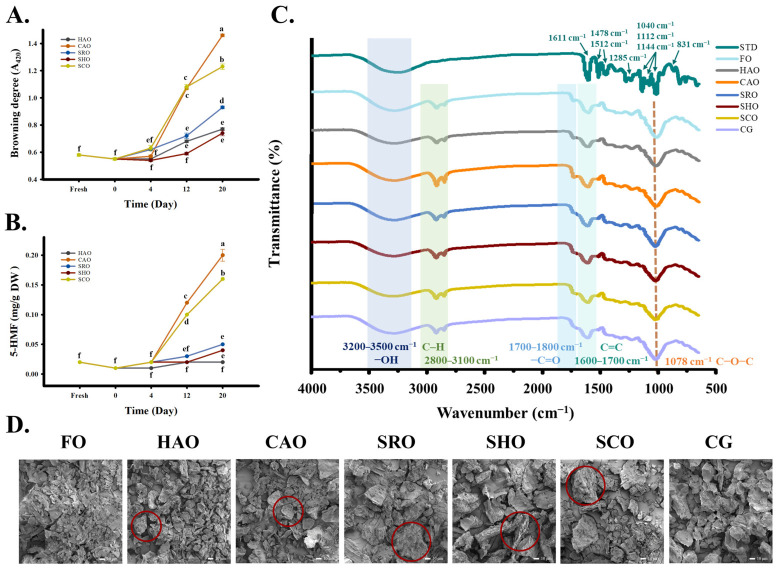
Browning characteristics of TKT under different SSA treatments, compared with commercial samples. (**A**) MRPs: browning degree (A_420_), (**B**) 5-HMF, (**C**) FTIR, and (**D**) FE-SEM. Different letters (a–f) indicate significant differences in the same group (*p* < 0.05). HAO, CAO, SRO, SHO, SCO, CG, and STD represent the hot-air drying, continuous aging, steam and room-temperature drying cycle, steam and hot-air drying cycle, and steam and constant temperature/humidity cycle SSA treatments, the commercial sample, and the catechin standard, respectively. 5-HMF, FTIR, and FE-SEM represent 5-hydroxymethylfurfural, Fourier-transform infrared spectroscopy, and field emission-scanning electron microscopy, respectively.

Previous studies have reported that Maillard reaction products exhibit antioxidant and anti-inflammatory properties [18,54]. The browning degree and 5-HMF content can serve as indicators for evaluating the efficiency of the Maillard reaction. The Maillard reaction imparts a dark brown appearance, attributed to the formation of intermediates and brown pigments, with a maximum absorption at 420 nm. The browning degree is positively correlated with the number of intermediate products formed [23,55]. Here, the browning degree of CAO and SCO increased, reaching 1.46 and 1.23, respectively. Thus, the high-humidity environment likely promoted the hydrolysis of disaccharides into glucose and fructose, providing more reactive substrates. During the SSA process, glucose may isomerize into fructose, which can subsequently undergo dehydration to form 5-HMF [56]. Therefore, CAO and SCO exhibited the highest 5-HMF contents, reaching 0.20 and 0.16 mg/g DW, respectively (Figure 2A).

The FTIR spectra and FE-SEM analyses were employed to examine changes in the internal structure and surface quality of the samples. The FTIR spectra revealed alterations in the functional groups during aging (Figure 2B). The broad band observed at 3200–3500 cm^−1^ corresponds to the stretching of -OH groups, characteristic of water, polyphenolic compounds, and hydroxyl-containing compounds. The 2800–3100 cm^−1^ region represents C–H stretching, where CAO showed a larger peak area. According to Hădărugă et al. [57], flavanone glycosides exhibit characteristic C–H stretching vibrations at 2982, 2907–2914, and 2876–2897 cm^−1^, which is consistent with the aforementioned increase in flavanone glycosides. The 1700–1800 cm^−1^ and 1600–1700 cm^−1^ regions correspond to carbonyl (-C=O) and aromatic (C=C) stretching, respectively. HAO exhibited smaller peak areas at 1600–1700 cm^−1^, suggesting partial cleavage of aromatic rings during aging, associated with decreased levels of alkenes, aldehydes, ketones, and esters [12]. The 950–1150 cm^−1^ region represents C-O-C stretching vibrations [13], indicative of the strength of glycosidic bonds in disaccharides. Larger peak areas in the FO and CG samples suggest a higher disaccharide content, whereas other groups subjected to different aging conditions displayed reduced peak areas as a result of disaccharide hydrolysis into monosaccharides, which subsequently served as substrates for the Maillard reaction [58]. Furthermore, a comparison with reference standards revealed characteristic catechin peaks at 831, 1040, 1112, 1144, 1285, 1478, 1512, and 1611 cm^−1^ [59]. Although FTIR provides macroscopic evidence of functional group alterations, it cannot definitively confirm specific intermolecular interactions such as π-π stacking, which typically requires NMR structural elucidation. Nevertheless, the observed spectral shifts, combined with the HPLC quantitative data demonstrating the release of aglycones and free phenolic acids, strongly support the hypothesis of glycosidic bond cleavage and the formation of stable polyphenol-flavanone complexes during the SSA process.

FE-SEM observations provided insights into the microstructure of TKT powder samples. Rapid moisture loss led to reduced porosity and compact surface structures in the hot-air-dried groups (HAO and SHO), with SHO showing the most pronounced shrinkage. This result can be attributed to steam treatment disrupting cell walls, followed by prolonged hot-air drying [60]. CAO exhibited partially loose or porous structures, where surface porosity could improve extraction efficiency and facilitate the release of bioactive compounds. The formation of porous structures is primarily due to cell wall softening and tissue relaxation under high temperature and humidity conditions [61]. By contrast, SRO and SCO developed wrinkled and less porous structures after steaming, as a result of the varying degrees of drying (Figure 2C).

### 3.4. Antioxidant Capacity and Blood Sugar Control Ability

**Figure 3:**
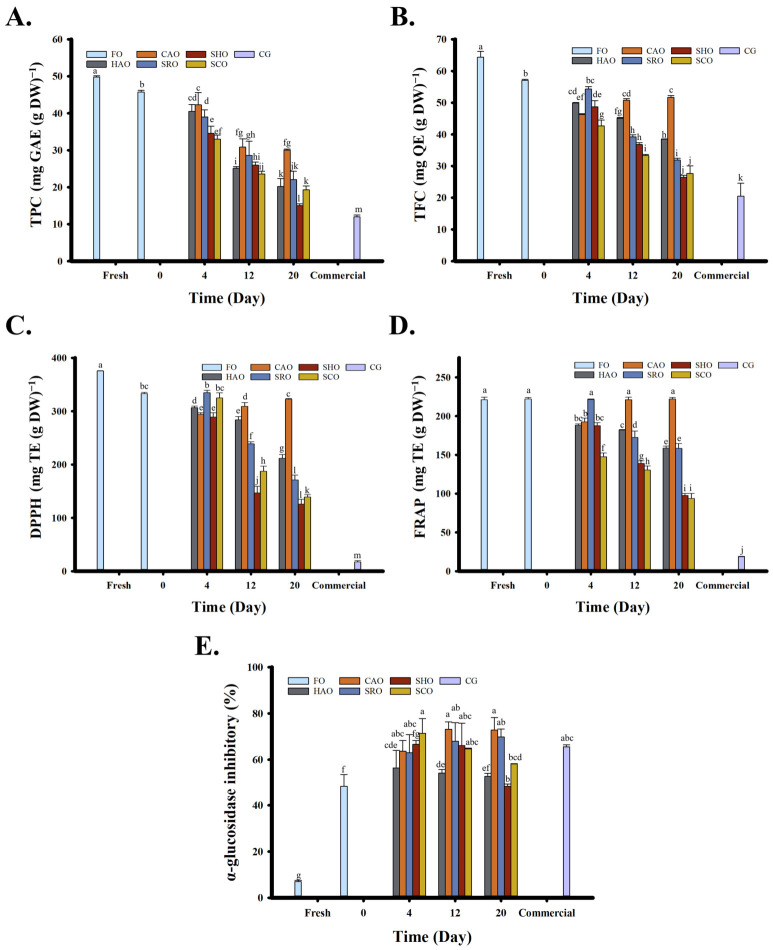
Antioxidant capacity and blood sugar control ability of TKT under different SSA treatments, compared with commercial samples. (**A**) Total polyphenol content (TPC); (**B**) total flavonoid content (TFC). Antioxidant capacity: (**C**) DPPH radical-scavenging activity; (**D**) ferric-reducing antioxidant power (FRAP); (**E**) α-glucosidase inhibitory activity. Data are presented as means ± standard deviations (*n* = 3). Different letters (a–m) indicate significant differences in the same group (*p* < 0.05). HAO, CAO, SRO, SHO, SCO, and CG represent the hot-air drying, continuous aging, steam and room-temperature drying cycle, steam and hot-air drying cycle, and steam and constant temperature/humidity cycle SSA treatments and the commercial sample, respectively. GAE, QE, and TE represent the gallic acid equivalent, quercetin equivalent, and Trolox equivalent, respectively.

Polyphenolic compounds are functional substances present in plant tissues. Numerous studies have highlighted their remarkable antioxidant, antidiabetic, and cardiovascular health-promoting properties [62,63]. Therefore, we performed polyphenol analysis as the basis for evaluating antioxidant capacity, which indicates the potential of TKT produced using different SSA treatments as functional foods. As shown in Figure 3A,B, the total polyphenol and total flavonoid contents (TPC and TFC) exhibited consistent trends. Fresh samples contained abundant polyphenols and flavonoids; however, their contents declined after processing, with TPC decreasing from 49.78 to 15.03–30.07 mg GAE/g DW, and total TFC decreasing from 64.35 to 26.43–51.69 mg QE/g DW. This decline can be attributed to thermal degradation, the spontaneous oxidation of polyphenols, and flavonoid breakdown [64]. However, compared to the commercial sample (CG), all SSA treatments retained higher phenolic contents, representing a 1.25- to 2.51-fold increase in TPC and a 1.29- to 2.52-fold increase in TFC. Notably, CAO exhibited the highest TPC and TFC after 20 days of SSA treatment, suggesting that the high temperature and humidity environment disrupted the plant cell walls, increased free polyphenol concentrations, and facilitated the hydrolysis of flavanone glycosides into flavonoids, resulting in higher concentrations, even after SSA [39,48].

Figure 3C,D presents the antioxidant capacity, expressed as Trolox equivalent antioxidant capacity (TEAC). In the DPPH radical scavenging assay, values declined from 375.32 to 125.94–211.90 mg TE/g DW, while the ferric-reducing antioxidant power decreased from 221.23 to 93.84–158.81 mg TE/g DW. Interestingly, CAO exhibited a slight increase in both assays, rising from 294.43 to 322.65 mg TE/g DW and from 192.70 to 222.02 mg TE/g DW, respectively, and performed better than the commercial sample. The SSA models exhibited 7.16- to 18.35-fold higher DPPH radical scavenging activity and 5.06- to 11.96-fold higher FRAP than the commercial product, which suffered severe activity losses because of uncontrolled repeated thermal processing. The overall decline in antioxidant activity could be ascribed to methodological limitations, as previously mentioned, where the contents of polymerized polyphenols might have been underestimated [65], along with thermal degradation of polyphenols reducing their apparent levels.

α-Glucosidase plays a critical role in postprandial blood glucose regulation, as it is located in the brush border of the small intestinal mucosa and functions as a key carbohydrate-hydrolyzing enzyme. Inhibiting α-glucosidase delays carbohydrate digestion, preventing postprandial hyperglycemia [17,66,67]. As shown in Figure 3E, inhibition rates significantly increased (*p* < 0.05) in all groups after processing, rising from 7.03% to 48.42–72.87%. Compared with FO, the inhibitory capacity increased at least 5.88–9.36 times, a change that can be attributed to the potent inhibitory effects of polyphenolic compounds such as catechins [20,21]. Previous studies have shown that the binding sites of eight catechin ligands (C, EC, GC, EGC, CG, ECG, GCG, EGCG) differ from that of acarbose. Polyphenols generally exhibit noncompetitive inhibition, and several catechin ligands have lower binding energies (C −7.5, EC −8.0, GC −8.4, EGC −8.0, CG −9.2, ECG −10.1, GCG −9.6, and EGCG −9.8 kcal/mol) than acarbose (−8.2 kcal/mol). Thus, they bind to α-glucosidase more effectively and stably and are more likely to promote inhibition [68,69]. Moreover, polyphenols may undergo oxidative cleavage during SSA to release free phenolic acids, providing more -OH groups to facilitate rapid binding with α-glucosidase [70,71]. In addition, recent studies have reported that 5-HMF possesses notable α-glucosidase inhibitory capacity [12,13]. Collectively, these findings demonstrate that SSA and their products can enhance α-glucosidase inhibition, highlighting their potential in the development of functional foods for glycemic regulation.

### 3.5. Overall Changes

The dashed lines in the AHC plot indicate statistical significance in the same group (*p* < 0.05). The data used in the analysis comprise the pH, total soluble solid (TSS), water activity in the peel (Aw-peel), water activity in the mixture (Aw-mixture), lightness (L*), browning degree (A_420_), 5-hydroxymethylfurfural (HMF), tartaric acid (TA), malic acid (MA), ascorbic acid (AA), narirutin, hesperidin, catechin, theaflavin, total polyphenol content (TPC), total flavonoid content (TFC), DPPH radical scavenging activity (DPPH), ferric-reducing antioxidant power (FRAP), and α-glucosidase inhibitory activity (α-GS). HAO, CAO, SRO, SHO, SCO, and CG represent the hot-air drying, continuous aging, steam and room-temperature drying cycle, steam and hot-air drying cycle, and steam and constant temperature/humidity cycle SSA treatments and the commercial sample, respectively.

**Figure 4:**
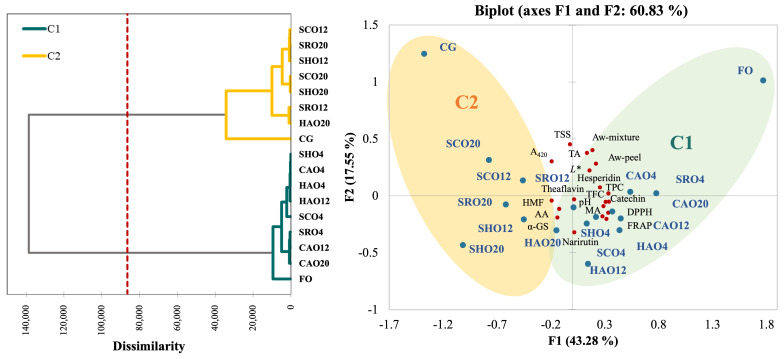
Agglomerative hierarchical clustering (AHC) and principal component analysis (PCA) of TKT under different SSA treatments, compared with the commercial sample.

We compiled experimental data from all SSA treatments and the commercial sample to better understand the changes in the physicochemical properties and functional components of the samples subjected to the different SSA treatments and shed light on the processing approaches of the commercial sample (Table S1). After normalization, we used AHC and PCA to determine the relationships among treatments. As shown in Figure 4, AHC divided the samples into two significantly (*p* < 0.05) distinct clusters: C1 (FO, HAO4, HAO12, CAO4, CAO12, CAO20, SRO4, SHO4, and SCO4) and C2 (HAO20, SRO12, SRO20, SHO12, SHO20, SCO12, SCO20, and CG). The PCA indicated that the representativeness of principal components F1 and F2 was 43.28% and 17.55%, respectively, accounting for a cumulative variance of 60.83%. On the right side of the PCA plot, C1 mainly comprised early-stage SSA groups and fresh samples, which exhibited higher TPC and TFC, as well as stronger antioxidant capacity. Notably, SSA-CAO provided a more favorable environment for promoting tea polyphenol oxidation–polymerization and Maillard reactions, generating abundant free phenolic acids, theaflavins, and Maillard reaction products that serve as efficient radical scavengers, resulting in superior antioxidant capacity [48,51]. By contrast, C2 included late-stage cyclic treatment groups and the commercial sample, suggesting that the processing of the commercial sample likely involves repeated steaming and heat-treatment cycles. This approach led to darker appearances but also substantial losses of functional components because of repeated thermal processing [43].

### 3.6. GC/MS Analysis

Citrus peel essential oil is rich in terpenoids, which are the main source of its strong aroma. During aging, small volatile substances, such as alcohols, aldehydes, ketones, acids, and esters, are derived from the Maillard reaction and other nonenzymatic browning or oxidative cleavage reactions. Therefore, exploring how different SSA treatments affect the aroma composition of TKT is essential. We analyzed the volatile components of TKT subjected to different SSA treatments (Table 3), and identified 106 volatile compounds: 15 alcohols, 10 aldehydes, 4 ketones, 3 acids, 6 esters, 4 ethers, 7 alkanes, 51 terpenes, 2 pyrroles, and 4 other components. Most of the aroma of TKT originates from terpenoids in citrus essential oils. High concentrations of monoterpenes were detected in multiple samples. Before aging, D-limonene and β-myrcene were dominant, and the samples presented a fresh citrus aroma accompanied by resin and herbal greenery notes [72]. Subsequently, monoterpenoid alcohols, such as linalool, α-terpineol, citronellol, geraniol, and geranyl acetate, increased with aging, giving the overall aroma a soft floral and elegant lilac note. This change softened the sharpness of the citrus peel essential oil, adding roundness and richness to the aroma [73]. In the later stages of aging, sesquiterpenes, such as nerolidol, δ-cadinene, α-copaene, γ-muurolene, α-amorphene, germacrene D, and β-bisabolene, imparted a mellow, woody, and spicy character, bringing out the rich, roasted aroma characteristic of teas that have undergone prolonged aging or roasting. The evolution from fresh fruity aromas and delicate floral notes to deep woody aromas results in profound and lingering sweetness during TKT aging [74].

However, in addition to the aforementioned characteristics of plant essential oils, compounds such as furans and pyrroles were also detected in the spectrum. These components are not typically found in unprocessed fresh plant tissues, but are products generated through the Maillard reaction or thermal degradation. They are commonly found in products that have undergone high-temperature roasting, fixation, or fermentation, forming distinctive aromas. In summary, TKT possesses both the fresh, volatile aroma derived from citrus raw materials and the mellow, roasted character imparted by the heat processing.

**Figure 5:**
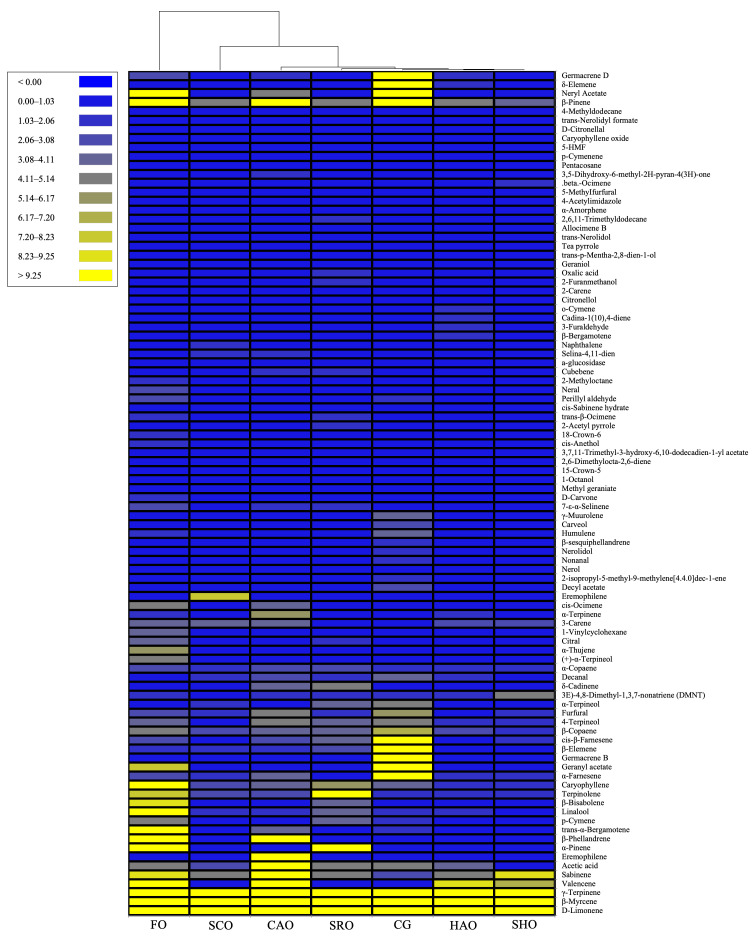
Heat maps of volatile compounds in TKT under different SSA treatments, compared with commercial samples.

In line with the preceding results, the heat map analysis showed that CAO exhibited the greatest antioxidant and anti-glycemic effects (Figure 5). Therefore, we performed volatile analysis to gain a deeper understanding of the changes in the bioactive composition of the samples. CAO showed a higher content of compounds 5, 28, 30, 50, 52, 54, 56, 57, 59, 63, 65, 80, 91, and 93, which are primarily terpenes and their oxygenated derivatives, than other SSA treatment groups (Table 3). Similarly, Hachlafi et al. [75] reported that orange essential oil is mainly composed of D-limonene, followed by thujene, myrcene, and α-pinene. These terpenes are excellent natural antioxidants. Furthermore, numerous studies have confirmed that the anti-glycemic mechanism is based on the synergistic action of three main pathways: inhibiting α-glucosidase, stimulating insulin secretion, and improving insulin resistance [76,77]. Based on the data from the current study and the literature, we speculate that the excellent antioxidant and anti-glycemic capabilities of CAO are due to its abundant terpenoid compounds.

### 3.7. Screening Potential α-Glucosidase Inhibitors and Docking Simulation

**Figure 6:**
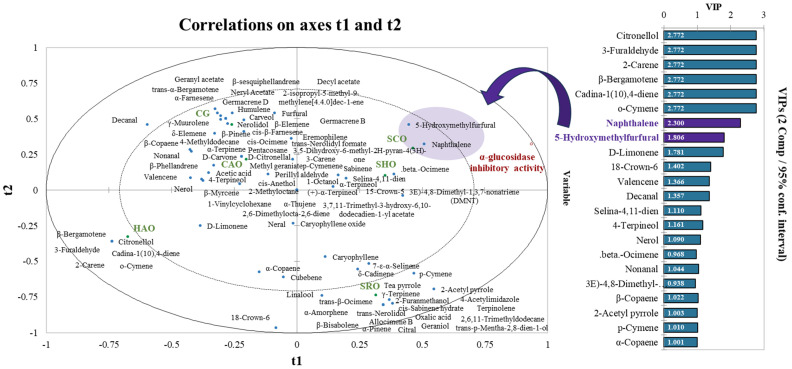
PLS-R loading plot and VIP scores illustrating the correlation between volatile compounds and α-glucosidase inhibitory activity.

We used the PLS-R model for correlation analysis. The variable importance in projection (VIP) value indicates the statistical contribution of a metabolite to the predictive model, with a value greater than 1 indicating a strong correlation with α-glucosidase inhibitory activity. Naphthalene (VIP = 2.300) and 5-hydroxymethylfurfural (VIP = 1.806) exhibited a high positive correlation (Figure 6) because of their distribution on the same side as the α-glucosidase inhibitory activity indicator. This result suggests that furan derivatives and aromatic compounds in TKT play a crucial role in inhibiting α-glucosidase.

The abundant monoterpenes (e.g., limonene and a-pinene) within the citrus peel undergo severe dehydrogenation and aromatization/cyclization under prolonged heating [78,79]. Its statistical collinearity with a-glucosidase inhibition is likely due to its synchronized formation alongside other process-induced metabolites (e.g., 5-HMF and polyphenols) during the SSA process. Given its well-documented toxicity, the presence of naphthalene serves strictly as a warning marker for excessive thermal degradation rather than a functional component. Its formation indicates the need for stringent temperature control during the SSA process to ensure food safety. Jung et al. [80] indicated that naphthyl glycosides can act as effective a-glucosidase inhibitors; however, the molecular interaction mechanism of a-glucosidase is currently unknown, and further in vivo toxicological evaluations are strictly required before considering any biological applications of these thermal byproducts.

**Figure 7:**
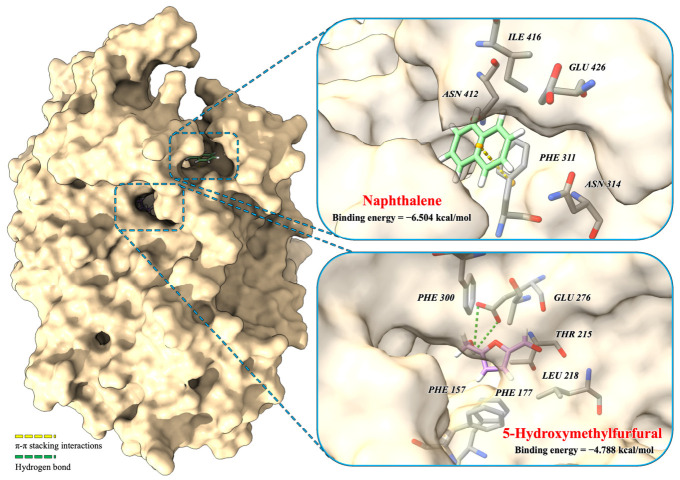
Molecular docking models of naphthalene and 5-hydroxymethylfurfural.

As shown in the simulation model in Figure 7, both 5-HMF and naphthalene bind efficiently to α-glucosidase, with binding energies of –4.788 and –6.504 kcal/mol, respectively. However, the two ligands bind to the acceptor in different ways. 5-HMF primarily exhibits competitive inhibition, using its oxygen atom to form a stable hydrogen bond with the acceptor GLU 276 [81]. Simultaneously, its furan ring structure generates hydrophobic interactions with surrounding aromatic amino acids, including PHE 300, PHE 157, and PHE 177. Naphthalene, on the other hand, exhibits noncompetitive inhibition, primarily through a stable π–π stacking interaction with PHE 311 and van der Waals forces with the surrounding ASN 412, ILE 416, and GLU 426.

This study confirms that the core mechanism of TKT in SSA lies in the biotransformation of large phytochemicals into small-molecule metabolites, which exhibit stronger free-radical scavenging ability and α-glucosidase affinity. During aging, the high temperature and high humidity environment provides kinetic energy, promoting the deconstruction of plant cell walls and the hydrolysis of glycosidic bonds, followed by a series of Maillard reactions that generate phytochemical metabolites with antioxidant potential and the ability to regulate postprandial blood glucose levels. Among them, 5-HMF, a potential inhibitor of α-glucosidase (binding energy −4.788 kcal/mol), increases the anti-glycemic potential of TKT through competitive inhibition. However, these binding modalities are based on computational simulations and require future *in vitro* enzyme kinetic studies for definitive experimental validation.

## 4. Conclusions

This study used solid-state aging (SSA) to regulate the biotransformation of Tsuan-Kan tea (TKT). The surface of the TKT tea rapidly browned within 8 days of aging (*ΔE* < 3.5). Continuous aging (CAO) at 55 °C and 75% relative humidity enhanced the antioxidant capacity and *in vitro* α-glucosidase inhibitory activity of TKT. This result was attributed to the initial deconstruction of the cell wall, which promoted the release of bioactive compounds leading to a sustained increase in the content of ascorbic acid, flavanone glycosides, and tea polyphenols by the fourth day of aging. Volatile compounds analysis showed that the aroma profile of TKT gradually evolved from initial fruity and floral aromas to woody and aged aromas. PLS-R and molecular docking analyses suggested that 5-HMF is a potential active component formed via nonenzymatic pathways. Although naphthalene exhibited high statistical correlation and theoretical binding affinity, it must strictly be regarded as a safety warning marker for excessive thermal degradation rather than a functional ingredient. Fixed-concentration *in vitro* assays were used as a preliminary screening of processing parameters to evaluate relative enzymatic inhibition. Although absolute IC_50_ values and cellular validations remain to be explored, this study demonstrated the chemical dynamics of TKT under different SSA models. The high-temperature and high-humidity environment of CAO facilitated the release of bioactive compounds and the nonenzymatic generation of 5-HMF. Moreover, this study highlights the necessity of monitoring thermal degradation byproducts, such as naphthalene, to ensure food safety. We will conduct further studies focused on the purification of specific active fractions, kinetic studies of IC_50_, and in vivo validations to bridge the gap between SSA technology and functional food development.

## Figures and Tables

**Table 1:** Physical properties of TKT under different SSA treatments, compared with the commercial sample.

Group	Day	pH	TSS (°Brix)	Water Activity	
				Peel	Mixture
FO	-	5.39 ± 0.01 ^a^	2.85 ± 0.07 ^b^	0.995 ± 0.000 ^a^	0.990 ± 0.000 ^a^
O0	0	5.01 ± 0.01 ^b^	2.75 ± 0.07 ^b^	0.711 ± 0.002 ^bc^	0.499 ± 0.001 ^i^
HAO	4	4.88 ± 0.01 ^d^	2.35 ± 0.07 ^de^	0.513 ± 0.004 ^fg^	0.420 ± 0.002 ^m^
	12	4.87 ± 0.00 ^d^	2.25 ± 0.07 ^ef^	0.385 ± 0.004 ^h^	0.268 ± 0.003 ^p^
	20	4.86 ± 0.01 ^e^	2.10 ± 0.00 ^gh^	0.329 ± 0.002 ^h^	0.254 ± 0.003 ^q^
CAO	4	4.90 ± 0.00 ^e^	2.55 ± 0.07 ^c^	0.744 ± 0.001 ^b^	0.684 ± 0.002 ^d^
	12	4.66 ± 0.01 ^h^	2.25 ± 0.07 ^ef^	0.716 ± 0.002 ^bc^	0.671 ± 0.002 ^e^
	20	4.56 ± 0.01 ^j^	2.15 ± 0.07 ^fgh^	0.640 ± 0.001 ^cde^	0.632 ± 0.001 ^g^
SRO	4	4.88 ± 0.01 ^h^	2.20 ± 0.00 ^fg^	0.574 ± 0.002 ^efg^	0.783 ± 0.002 ^b^
	12	4.59 ± 0.01 ^i^	2.45 ± 0.07 ^cd^	0.563 ± 0.001 ^efg^	0.639 ± 0.002 ^f^
	20	4.38 ± 0.01 ^l^	2.60 ± 0.00 ^c^	0.413 ± 0.002 ^h^	0.387 ± 0.002 ^n^
SHO	4	4.80 ± 0.00 ^f^	2.55 ± 0.07 ^c^	0.637 ± 0.003 ^cde^	0.367 ± 0.001 ^o^
	12	4.52 ± 0.01 ^k^	2.05 ± 0.07 ^h^	0.522 ± 0.002 ^fg^	0.423 ± 0.002 ^l^
	20	4.26 ± 0.00 ^n^	2.05 ± 0.07 ^h^	0.337 ± 0.002 ^h^	0.217 ± 0.002 ^r^
SCO	4	4.72 ± 0.00 ^g^	2.45 ± 0.07 ^cd^	0.667 ± 0.002 ^bcd^	0.453 ± 0.001 ^k^
	12	4.33 ± 0.01 ^m^	2.75 ± 0.07 ^b^	0.500 ± 0.210 ^g^	0.483 ± 0.002 ^j^
	20	4.14 ± 0.00 ^o^	2.45 ± 0.07 ^cd^	0.604 ± 0.002 ^def^	0.608 ± 0.002 ^h^
CG	-	3.86 ± 0.01 ^p^	3.17 ± 0.15 ^a^	0.567 ± 0.002 ^efg^	0.688 ± 0.002 ^c^

Data are presented as means ± standard deviations (*n* = 3). Different letters (a–r) indicate significant differences in the same group (*p* < 0.05). HAO, CAO, SRO, SHO, SCO, and CG represent the hot-air drying, continuous aging, steam and room-temperature drying cycle, steam and hot-air drying cycle, steam and constant temperature/humidity cycle SSA treatments and commercial sample, respectively. TSS means total soluble solids.

**Table 2:** Analysis of organic acids and polyphenols in TKT subjected to different SSA treatments, compared with a commercial sample.

Group	Day	Organic Acids (mg/g DW)			Polyphenols (mg/g DW)			
		Tartaric Acid	Malic Acid	Ascorbic Acid	Narirutin	Hesperidin	Catechins	Theaflavin
FO	-	7.56 ± 0.59 ^a^	1.96 ± 0.00 ^bc^	0.76 ± 0.06 ^j^	1.90 ± 0.01 ^h^	41.54 ± 0.14 ^a^	117.87 ± 0.95 ^a^	1.78 ± 0.00 ^b^
O0	0	6.47 ± 0.01 ^b^	2.14 ± 0.00 ^ab^	0.79 ± 0.00 ^j^	2.94 ± 0.02 ^a^	15.59 ± 0.28 ^d^	101.11 ± 1.38 ^g^	1.70 ± 0.00 ^i^
HAO	4	5.29 ± 0.15 ^cde^	1.69 ± 0.03 ^de^	0.82 ± 0.06 ^ij^	2.68 ± 0.01 ^c^	ND	101.75 ± 0.20 ^fg^	1.70 ± 0.00 ^h^
	12	2.74 ± 0.00 ^i^	2.03 ± 0.05 ^abc^	0.78 ± 0.00 ^j^	2.59 ± 0.00 ^d^	4.15 ± 0.00 ^gh^	96.89 ± 0.44 ^i^	1.68 ± 0.00 ^k^
	20	3.38 ± 0.14 ^hi^	2.01 ± 0.12 ^bc^	1.06 ± 0.02 ^bc^	1.61 ± 0.00 ^i^	29.19 ± 0.91 ^c^	90.11 ± 0.86 ^l^	1.69 ± 0.00 ^j^
CAO	4	4.39 ± 0.67 ^d^	2.10 ± 0.01 ^ab^	1.08 ± 0.02 ^h^	1.59 ± 0.00 ^i^	ND	102.03 ± 0.20 ^fg^	1.68 ± 0.00 ^l^
	12	4.33 ± 0.27 ^fg^	2.26 ± 0.01 ^a^	1.10 ± 0.02 ^h^	2.18 ± 0.00 ^g^	8.34 ± 0.61 ^f^	108.40 ± 0.99 ^d^	1.73 ± 0.00 ^f^
	20	3.57 ± 0.07 ^gh^	1.81 ± 0.10 ^cd^	1.00 ± 0.02 ^cde^	2.81 ± 0.01 ^b^	37.33 ± 2.52 ^b^	109.80 ± 1.41 ^c^	1.82 ± 0.00 ^a^
SRO	4	5.59 ± 0.10 ^cd^	1.99 ± 0.07 ^bc^	0.92 ± 0.04 ^fgh^	1.45 ± 0.01 ^j^	ND	111.38 ± 0.27 ^b^	1.75 ± 0.00 ^c^
	12	4.18 ± 0.49 ^fg^	1.50 ± 0.32 ^ef^	0.96 ± 0.01 ^def^	2.48 ± 0.15 ^e^	3.77 ± 2.13 ^h^	103.78 ± 0.31 ^e^	1.74 ± 0.00 ^e^
	20	2.69 ± 0.07 ^i^	0.96 ± 0.23 ^h^	0.98 ± 0.00 ^efg^	2.11 ± 0.00 ^g^	10.68 ± 1.90 ^e^	94.60 ± 0.18 ^j^	1.70 ± 0.00 ^h^
SHO	4	4.95 ± 0.17 ^def^	1.37 ± 0.00 ^fg^	0.95 ± 0.02 ^efg^	2.41 ± 0.00 ^e^	ND	102.96 ± 0.18 ^ef^	1.74 ± 0.00 ^d^
	12	4.67 ± 0.28 ^ef^	1.22 ± 0.01 ^g^	0.91 ± 0.01 ^gh^	2.10 ± 0.01 ^g^	ND	91.71 ± 0.13 ^k^	1.69 ± 0.00 ^j^
	20	3.12 ± 0.90 ^hi^	1.51 ± 0.09 ^fg^	1.04 ± 0.02 ^bcd^	2.17 ± 0.02 ^g^	ND	78.98 ± 0.46 ^m^	1.62 ± 0.00 ^n^
SCO	4	4.69 ± 0.13 ^ef^	1.61 ± 0.03 ^def^	0.99 ± 0.06 ^de^	2.57 ± 0.01 ^d^	ND	98.62 ± 0.28 ^h^	1.71 ± 0.00 ^g^
	12	5.84 ± 0.43 ^bc^	1.59 ± 0.03 ^def^	1.01 ± 0.02 ^cde^	2.69 ± 0.00 ^c^	6.06 ± 0.54 ^g^	91.77 ± 0.00 ^k^	1.64 ± 0.00 ^m^
	20	5.53 ± 0.06 ^cd^	1.37 ± 0.11 ^fg^	1.20 ± 0.01 ^a^	2.29 ± 0.00 ^f^	17.50 ± 0.64 ^d^	91.79 ± 0.05 ^k^	1.62 ± 0.00 ^o^
CG	-	5.42 ± 0.02 ^cde^	0.94 ± 0.00 ^h^	0.86 ± 0.02 ^hi^	1.34 ± 0.00 ^k^	3.50 ± 0.57 ^h^	71.36 ± 0.26 ^n^	ND

Data are presented as means ± standard deviations (*n* = 3). Different letters (a–o) indicate significant differences in the same group (*p* < 0.05). ND means not detected (lower than the limit of quantification, LOQ). HAO, CAO, SRO, SHO, SCO, and CG represent the hot-air drying, continuous aging, steam and room-temperature drying cycle, steam and hot-air drying cycle, steam and constant temperature/humidity cycle SSA treatments, and the commercial sample, respectively.

**Table 3:** Analysis of volatile compounds in TKT under different SSA treatments, compared with commercial samples.

No.	RI	Compound	Formula	Scent	Compositions (μg/g)						
					FO	HAO	CAO	SRO	SHO	SCO	CG
Alcohol											
1	885	2-Furanmethanol	C_5_H_6_O_2_	Burnt, Caramel, Coffee	–	–	–	1.69	–	–	–
2	1041	*cis*-Sabinene hydrate	C_10_H_18_O	Balsamic, Herbal, Earthy	1.00	–	–	0.83	–	–	–
3	1059	1-Octanol	C_8_H_18_O	Waxy, Green, Citrus	0.70	–	–	–	–	–	–
4	1082	Linalool	C_10_H_18_O	Floral, Lavender, Citrus	9.25	1.36	1.42	3.33	0.78	1.04	1.57
5	1137	4-Terpineol	C_10_H_18_O	Earthy, Pepper, Woody	3.80	1.57	4.66	3.33	1.54	–	4.65
6	1140	*trans*-*p*-Mentha-2,8-dien-1-ol	C_10_H_18_O	Minty, Spicy, Oily, Herbal	–	–	–	0.53	–	–	–
7	1143	(+)-α-Terpineol	C_10_H_18_O	Lilac, Pine, Soapy	4.50	–	–	–	–	–	–
8	1147	α-Terpineol	C_10_H_18_O	Lilac, Sweet, Floral	–	–	–	3.40	0.90	1.04	4.80
9	1179	Citronellol	C_10_H_18_O	Rose, Citrus, Waxy	–	0.68	–	–	–	–	–
10	1206	*cis*-Carveol	C_10_H_16_O	Spearmint, Caraway, Herbal	–	–	–	0.32	–	–	–
11	1208	Carveol	C_10_H_16_O	Spearmint, Spicy	–	–	–	–	–	–	3.04
12	1228	Nerol	C_10_H_18_O	Rose, Citrus, Fresh	0.69	0.24	–	0.33	–	–	0.79
13	1234	Geraniol	C_10_H_18_O	Sweet Rose, Fruity	–	–	–	0.54	–	–	–
14	1564	Nerolidol	C_15_H_26_O	Floral, Woody, Waxy	–	–	–	–	–	–	1.26
15	1566	*trans*-Nerolidol	C_15_H_26_O	Woody, Floral, Apple skin	–	–	–	0.67	–	–	–
Aldehydes											
16	831	Furfural	C_5_H_4_O_2_	Bread, Almond, Woody	–	–	–	–	–	–	–
17	835	3-Furaldehyde	C_5_H_4_O_2_	Grain, Toasted	–	1.48	–	–	–	–	–
18	920	5-Hydroxymethylfurfural	C_6_H_6_O_2_	Caramel, Burnt sugar	–	–	0.52	–	0.56	0.38	–
19	982	Benzaldehyde	C_7_H_6_O	Bitter almond, Cherry	–	–	–	0.30	–	–	–
20	1104	Nonanal	C_9_H_18_O	Waxy, Citrus Peel	–	0.34	–	0.26	–	–	1.37
21	1125	D-Citronellal	C_10_H_18_O	Citrus, Green, Rose	–	–	0.64	–	–	–	–
22	1174	Neral	C_10_H_16_O	Lemon, Sweet	2.14	–	–	–	–	–	–
23	1176	Citral	C_10_H_16_O	Lemon, Fresh, Citrus Peel	3.90	–	–	1.28	–	–	–
24	1204	Decanal	C_10_H_20_O	Citrus, Waxy, Floral	–	1.85	2.89	1.42	0.88	1.25	3.72
25	1207	Perillyl aldehyde	C_10_H_14_O	Spicy, Woody, Citrus	2.27	–	–	0.53	–	–	1.09
Ketones											
26	698	Hydroxyacetone	C_3_H_6_O_2_	Pungent, Caramel, Sweet	–	–	0.48	–	–	–	–
27	1190	D-Carvone	C_10_H_14_O	Caraway, Spearmint, Herbal	1.34	–	0.93	–	–	–	–
28	1269	3,5-Dihydroxy-6-methyl-2H-pyran-4(3H)-one	C_6_H_8_O_4_	Caramel, Burnt sugar	–	–	1.17	–	–	–	–
29	1457	β-Lonone	C_13_H_20_O	Violet, Raspberry, Woody	–	–	0.30	–	–	–	–
Acids											
30	576	Acetic acid	C_2_H_4_O_2_	Sour, Vinegar, Pungent	4.17	3.52	15.20	4.93	–	2.22	5.01
31	933	Oxalic acid	C_2_H_2_O_4_	Acidic	–	–	–	1.59	–	–	–
32	1752	*trans*-Nerolidyl formate	C_16_H_26_O_2_	Woody, Floral, Waxy	–	–	0.56	–	–	–	–
Ester											
33	1183	Octyl acetate	C_10_H_20_O_2_	Fruity, Waxy	–	0.36	–	0.42	–	–	–
34	1252	Methyl geraniate	C_11_H_18_O_2_	Waxy, Green, Floral	0.79	–	0.56	–	–	–	–
35	1352	Neryl Acetate	C_12_H_20_O_2_	Floral, Raspberry, Fruity	12.13	–	4.47	–	–	–	35.94
36	1352	Geranyl acetate	C_12_H_20_O_2_	Rose, Lavender, Fruity	7.98	–	–	–	–	–	11.35
37	1381	Decyl acetate	C_12_H_24_O_2_	Rose, Waxy, Fruity	–	–	–	–	–	–	2.06
38	1940	3,7,11-Trimethyl-3-hydroxy-6,10-dodecadien-1-yl acetate	C_17_H_30_O_3_	Faint, Waxy, Balsamic	0.97	–	–	–	–	–	–
Ethers											
39	1186	*cis*-Anethol	C_10_H_12_O	Anise, Licorice, Sweet	1.19	–	–	–	–	–	–
40	1507	Caryophyllene oxide	C_15_H_24_O	Woody, Dry, Amber	–	–	0.65	0.39	–	–	–
41	1744	15-Crown-5	C_10_H_20_O_5_	Chemical, Solvent	0.64	–	–	–	–	–	–
42	2092	18-Crown-6	C_12_H_24_O_6_	Chemical, Solvent	1.63	0.43	–	0.73	–	–	–
Alkanes											
43	852	2-Methyloctane	C_9_H_20_	Petrol, Alkane, Mild	1.64	–	–	–	–	–	–
44	870	1-Vinylcyclohexane	C_12_H_26_	Gassy, Chemical	3.25	–	–	–	–	–	–
45	1214	Dodecane	C_12_H_26_	Waxy, Clean, Mild	–	–	–	–	–	0.27	–
46	1256	4-Methyldodecane	C_13_H_28_	Waxy, Faint	–	–	0.55	–	–	–	–
47	1320	2,6,11-Trimethyldodecane	C_15_H_32_	Waxy, Odorless	–	–	–	1.17	–	–	–
48	1413	Tetradecane	C_14_H_30_	Waxy, Mild	–	–	–	0.42	–	–	–
49	2506	Pentacosane	C_25_H_52_	Waxy, Odorless	–	–	0.87	–	–	–	–
Terpenes											
50	897	Sabinene	C_10_H_16_	Woody, Spicy, Pepper	8.41	4.17	10.99	4.90	8.83	4.70	2.45
51	902	α-Thujene	C_10_H_16_	Woody, Green, Herbal	5.43	–	–	–	–	–	–
52	943	β-Pinene	C_10_H_16_	Pine, Resinous, Green	36.03	4.87	42.34	4.15	3.86	4.61	16.30
53	948	α-Pinene	C_10_H_16_	Pine, Earthy, Fresh	13.81	–	–	22.81	–	–	–
54	950	3-Carene	C_10_H_16_	Sweet, Pine, Resinous	3.80	2.95	4.00	0.55	2.49	3.62	–
55	952	2-Carene	C_10_H_16_	Pine, Sweet	–	0.82	–	–	–	–	–
56	958	β-Myrcene	C_10_H_16_	Peppery, Balsamic, Spice	74.57	38.57	77.79	40.60	28.29	37.57	30.22
57	964	β-Phellandrene	C_10_H_16_	Minty, Pepper, Citrus	10.21	–	10.08	–	–	–	–
58	972	*trans*-β-Ocimene	C_10_H_16_	Green, Tropical, Floral	1.18	–	–	1.06	–	–	–
59	976	*cis*-Ocimene	C_10_H_16_	Floral, Herbal, Green	4.51	–	3.18	–	–	–	–
60	982	β-Ocimene	C_10_H_16_	Green, Tropical, Woody	–	–	–	–	1.05	–	–
61	985	2,6-Dimethylocta-2,6-diene	C_10_H_18_	Citrus, Green	0.59	–	–	–	–	–	–
62	993	Allocimene B	C_10_H_16_	Herbal, Floral	–	–	–	0.60	–	–	–
63	996	α-Terpinene	C_10_H_16_	Lemon, Woody, Herbal	1.91	1.12	5.31	0.06	0.79	–	–
64	998	γ-Terpinene	C_10_H_16_	Lime, Sweet	96.97	10.13	9.90	98.45	13.09	14.00	17.14
65	1018	D-Limonene	C_10_H_16_	Citrus, Fresh	1327.37	754.10	914.95	833.18	810.54	450.38	791.56
66	1023	3-Isopropylidene-6-methyl-cyclohexene	C_15_H_24_	Herbal, Green	–	–	–	0.35	–	–	–
67	1042	*p*-Cymene	C_10_H_14_	Citrus, Solvent, Spice	4.85	–	0.77	4.02	0.67	0.99	1.32
68	1046	*o*-Cymene	C_10_H_14_	Solvent, Harsh	–	1.04	–	–	–	–	–
69	1052	Terpinolene	C_10_H_16_	Pine, Citrus, Floral	8.08	1.76	2.06	9.41	1.75	2.27	1.58
70	1056	*p*-Cymenene	C_10_H_16_	Woody, Spicy, Herbal	–	–	0.92	–	–	–	–
71	1075	3E)-4,8-Dimethyl-1,3,7-nonatriene (DMNT)	C_10_H_16_	Green, Herbal	1.46	1.12	1.24	1.90	4.64	0.95	1.39
72	1125	Cyclosativene	C_15_H_24_	Woody, Herbal	–	–	–	0.42	–	–	–
73	1216	β-Copaene	C_15_H_24_	Woody, Spicy	4.16	2.99	3.15	3.36	1.99	2.12	6.64
74	1221	α-Copaene	C_15_H_24_	Woody	2.52	1.83	2.79	3.00	1.04	1.30	1.52
75	1339	Cubebene	C_15_H_24_	Spicy, Herbal, Woody	0.87	0.93	1.44	1.33	0.44	0.60	–
76	1377	δ-Elemene	C_15_H_24_	Woody, Floral	–	1.28	–	–	–	–	50.70
77	1398	β-Elemene	C_15_H_24_	Woody, Waxy, Sweet	1.85	–	1.94	2.17	–	2.02	15.78
78	1403	α-Maaliene	C_15_H_24_	Woody	–	–	–	–	0.43	–	–
79	1411	β-Panasinsene	C_15_H_24_	Herbal, Woody	–	–	–	0.36	–	–	–
80	1428	*trans*-α-Bergamotene	C_15_H_24_	Citrus, Woody, Green	12.19	–	3.29	–	–	0.64	1.50
81	1430	Bergamotene	C_15_H_24_	Citrus, Green	–	–	–	0.35	–	–	–
82	1435	γ-Muurolene	C_15_H_24_	Woody, Spicy, Herbal	–	–	–	–	–	–	3.60
83	1440	β-Bergamotene	C_15_H_24_	Citrus, Woody	–	1.26	–	–	–	–	–
84	1442	*cis*-β-Farnesene	C_15_H_24_	Woody, Herbal	2.80	–	2.46	3.18	1.22	0.39	13.89
85	1445	α-Amorphene	C_15_H_24_	Woody, Spicy	–	–	–	0.90	–	–	–
86	1446	β-sesquiphellandrene	C_15_H_24_	Woody, Green	–	–	–	–	–	–	1.13
87	1458	α-Farnesene	C_15_H_24_	Green Apple, Floral, Woody	2.22	1.49	3.09	–	1.49	1.29	22.32
88	1464	2-isopropyl-5-methyl-9-methylene[4.4.0]dec-1-ene	C_15_H_24_	Woody	–	–	–	–	–	–	1.88
89	1469	δ-Cadinene	C_15_H_24_	Thyme, Woody, Medicinal	–	–	3.28	4.29	0.96	–	–
90	1474	Valencene	C_15_H_24_	Citrus, Woody	19.44	8.70	19.10	0.63	6.78	–	–
91	1476	7-ε-α-Selinene	C_15_H_24_	Amber, Woody	2.29	–	1.30	1.76	–	0.44	–
92	1482	Eremophilene	C_15_H_24_	Woody, Warm	–	–	19.20	–	–	7.50	–
93	1490	α-Guaiene	C_15_H_24_	Woody, Balsamic, Earthy	–	–	–	0.45	–	–	–
94	1494	Caryophyllene	C_15_H_24_	Pepper, Spicy, Woody	11.61	1.99	3.67	6.15	1.83	2.08	4.02
95	1500	β-Bisabolene	C_15_H_24_	Balsamic, Citrus, Woody	9.19	–	–	3.18	–	–	–
96	1502	Selina-4,11-dien	C_15_H_24_	Woody	–	0.80	1.34	0.65	–	2.05	–
97	1515	Germacrene D	C_15_H_24_	Woody, Spicy, Earthy	2.56	1.13	1.96	–	0.65	0.72	42.81
98	1579	Humulene	C_15_H_24_	Hops, Woody, Earthy	1.33	0.37	0.91	0.81	–	0.68	4.03
99	1603	Germacrene B	C_15_H_24_	Green, Woody, Fresh	–	–	–	–	–	–	9.52
100	1766	Cadina-1,4-diene	C_15_H_24_	Woody, Herbal	–	1.42	–	–	–	–	–
Pyrrole											
101	1035	2-Acetyl pyrrole	C_6_H_7_NO	Nutty, Roasted, Bread	0.95	–	–	1.07	0.65	–	–
102	1054	Tea pyrrole	C_7_H_9_NO	Roasted Tea, Burnt, Nutty	–	–	–	0.53	–	–	–
Other											
103	1072	4-Acetylimidazole	C_5_H_6_N_2_O	Roasted, Chemical	–	–	–	0.96	–	–	–
104	1470	Naphthalene	C_10_H_8_	Mothball, Tar, Chemical	–	–	–	–	–	1.44	–
105	1628	1-Iodododecane	C_12_H_25_I	Chemical, Waxy	–	–	0.46	–	–	–	–
106	3219	1-Iododotriacontane	C_28_H_57_I	Chemical, Waxy	–	–	–	0.44	–	–	–

HAO, CAO, SRO, SHO, SCO, and CG represent the hot-air drying, continuous aging, steam and room-temperature drying cycle, steam and hot-air drying cycle, and steam and constant temperature/humidity cycle SSA treatments and the commercial sample, respectively. “–” and RI mean not detected and retention index, respectively. The scent type corresponded with information from The Good Scents Company Information System. HAO, CAO, SRO, SHO, SCO, and CG represent the hot-air drying, continuous aging, steam and room-temperature drying cycle, steam and hot-air drying cycle, and steam and constant temperature/humidity cycle SSA treatments and the commercial sample, respectively.

## Data Availability

The original contributions presented in this study are included in the article/Supplementary Materials. Further inquiries can be directed to the corresponding author.

## Supplementary Materials

The following supporting information can be downloaded at: https://www.mdpi.com/article/10.3390/foods15142535/s1, Figure S1: Calibration curve of standards; Table S1: Physical properties of commercial sample.
