# Supplementary material for: α-Glucosidase Inhibitory Activity of Tsuan-Kan Tea in Different Solid-State Aging Models: Phytochemical Biotransformation, Volatile Profile, and Molecular Docking
Source: Foods. 2026 Jul 17;15(14):2535. doi: 10.3390/foods15142535 (PMC13407562; doi:10.3390/foods15142535)
Supplement: Supplementary file 1 [file foods-15-02535-s001.zip › foods-4404466-supplementary.pdf]

Supplementary Materials: The following supporting information can be downloaded at:

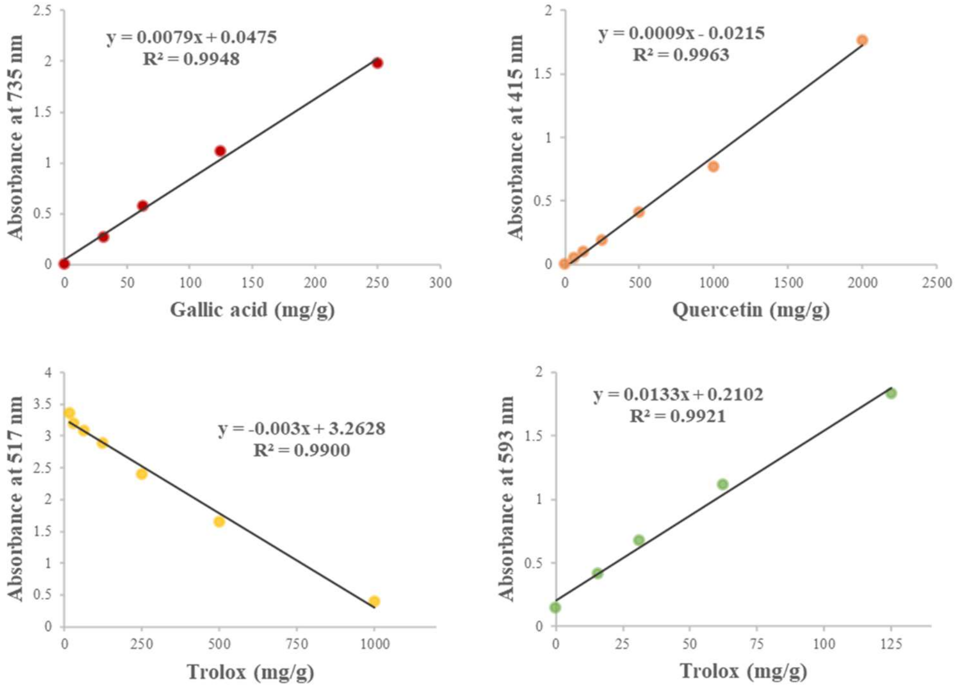

Figure S1. Calibration curve of standards.

Table S1. Physical properties of commercial sample.

	Weight (g)	Aspect ratio	*L**	*a**	*b**	A_{420}	5-HMF (mg/g DW)
**CG**	236.53 ± 0.00	0.48 ± 0.00	12.49 ± 0.00	0.87 ± 0.06	0.56 ± 0.02	2.05 ± 0.04	ND

Data are presented as means ± standard deviations (*n* = 3). ND represents not detected (Lower than LOQ). CG is commercial sample.
